# No effect of recumbency duration on the occurrence of post-lumbar puncture headache with a 22G cutting needle

**DOI:** 10.1186/1471-2377-12-1

**Published:** 2012-01-30

**Authors:** Sung R Kim, Hyun S Chae, Mi J Yoon, Jung H Han, Kwang J Cho, Sun J Chung

**Affiliations:** 1 Department of Nursing, Asan Medical Center, Seoul, South Korea; 2 Department of Neurology, Asan Medical Center, University of Ulsan College of Medicine, Seoul, South Korea

## Abstract

**Background:**

Supine recumbence has been widely performed to prevent post-lumbar puncture headache (PLPH). However, the optimal duration of supine recumbence is controversial. The aim of the study is to compare the occurrence of PLPH according to the duration of supine recumbence in patients with neurological disorders.

**Methods:**

A non-equivalent control/experimental pre-/post-test study design was used. Seventy consecutive patients were prospectively enrolled between July 2007 and July 2008. Thirty-five patients underwent supine recumbence for four hours after lumbar puncture (Group 1) and 35 patients underwent supine recumbence for one hour (Group 2).

**Results:**

The overall frequency of PLPH was 31.4%. The frequency of PLPH was not significantly different between the Group 1 (28.6%) and Group 2 (34.3%) (P = 0.607). In patients with PLPH, the median severity (P = 0.203) and median onset time of PLPH (P = 0.582) were not significantly different between the two groups. In a logistic regression analysis, the previous history of post-lumbar puncture headache was a significant risk factor for the occurrence of PLPH (OR = 11.250, 95% CI: 1.10-114.369, P = 0.041).

**Conclusions:**

Our study suggests that short duration (one hour) of supine recumbence may be as efficient as long duration (four hours) of supine recumbence to prevent PLPH.

## Background

Lumbar puncture (LP) is an essential medical procedure in many clinical practices. Although LP is relatively safe, several adverse events have been reported, including headache, hemorrhage, local pain, and infections[1]. Among these adverse events, post-lumbar puncture headache (PLPH) is the most frequent and disabling in many patients. The International Headache Society has defined a PLPH as a headache that develops within 5 days of dural puncture and resolves within 1 week spontaneously or within 48 hours after effective treatment of the spinal fluid leak (usually by epidural blood patch)[2]. The headache usually worsens within 15 minutes after sitting or standing, and disappears or improves within 15 minutes after lying down. The headache is generally located in the frontal or occipital areas, or both, but may also involve the neck and upper shoulders. The severe PLPH may be associated with nausea, vomiting, blurred vision, vertigo, hearing alteration, and back pain[3].

PLPH has been considered to result from CSF leakage through the dural tear into the epidural space. A large hole in the dura leads to a greater CSF volume loss, which increases the odds of developing PLPH. Needle size may be the most important factor in determining the risk of PLPH[4]. Other procedure-related risk factors include needle shape, needle orientation, stylet reinsertion, and operator experience[5-7]. Other protective factors bed rest, hydration, patient positioning during the LP, the volume of CSF removed during dural puncture, as well as the number of attempts have not been shown to be associated with reduced PLPH incidence[3,8,9]. Two studies showed slightly increased frequency of PLPH in recumbent patients as compared with patients immediately immobilized, while one study showed no significant difference in prevalence, pattern and severity of PLPH between 6 hours of recumbence and early ambulation. However, there are serious methodologic concerns regarding these previous studies. Therefore, the strict bed rest after LP for variable time period is still advised by many physicians.

In this scope of current evidence, we aimed to prospectively evaluate the difference in the occurrence rate of PLPH between patients who had 1 hour of supine recumbence and those who had 4 hours of supine recumbence.

## Methods

### Study design

A non-equivalent control/experimental pre/post study design was used. Between July 1, 2007 and December 15, 2007, all consecutive patients who underwent LP had supine recumbence for 4 hours after LP (Group 1, n = 35). Between January 15, 2008 and July 31, 2008, all patients who underwent LP had supine recumbence for 1 hour after LP (Group 2, n = 35).

### Subjects

All 70 subjects were prospectively recruited from Neurology clinic of the Asan Medical Center, Seoul, Korea. The PLPH was defined according to the definition suggested by The International Headache Society: 1) A headache that develops within 5 days of dural puncture and resolves within 1 week; 2) The headache usually worsens within 15 minutes after sitting or standing, and disappears or improves within 15 minutes after lying down[2]. The inclusion criteria were as follows: 1) PLPH patients > 18 years of age with neurological disorders, 2) PLPH patients without cognitive impairment, 3) PLPH patients who underwent LP using a 22-gauge cutting needle, and 4) PLPH patients who underwent LP for diagnosis but not for treatment. The exclusion criteria were 1) patients who changed analgesics on the day of LP, 2) patients who underwent LP using a needle other than 22-gauge cutting needle, 3) patients with spontaneous CSF leakage, 4) patients who underwent CSF drainage (especially in cases of normal pressure hydrocephalus).

### Procedure

The LP was performed by a physician under the standardized institutional guideline for LP. The bevel direction of the cutting needle was parallel to the dural fibers and the long axis of the spine. The severity of headache was assessed before LP and after completion of supine recumbence using a Numeric Rating Scale (NRS). To assess postural headache after supine recumbence, all patients were asked to stand for 15 minutes. For patients with postural headache after supine recumbence, the headache was assessed every 8 hours until the postural headache resolved. A 0 to 10-point NRS was chosen, with an endpoint of extreme pain and a zero point of no pain.

We collected demographic and clinical characteristics, including disease diagnosis, CSF profiles, CSF pressure, CSF drainage volume, number of LP attempts, experience of chronic headache, experience of LP, experience of PLPH, and treatment using analgesics after PLPH. Chronic headache was defined in this study as a headache on 15 or more days per month. In addition, chronic headache had been occurred and was present before a LP with constant severity of headache and no relationship with changes of body position.

### Statistical Analysis

The appropriate sample size of 70 subjects (35 in an experimental group and 35 in a control group) was calculated by power analysis, with a level of significance of 0.05, a power of 80%, and a large effect size. Data were analyzed using SPSS version 11.5 (SPSS Inc., Chicago, IL) to obtain descriptive statistics for all variables. Differences between Group 1 and Group 2 patients with respect to demographic and clinical characteristics were assessed by the chi-square test, Fisher's exact test, the t-test, and ANOVA. The difference in PLPH frequency between Groups 1 and 2 was assessed by the chi-square test. A P-value of < 0.05 was considered statistically significant.

### Ethical considerations

This study was approved by the Institutional Review Board (IRB) of the Asan Medical Center. We obtained written informed consent from all patients after a nurse had explained the aims and procedures of the study.

## Results

### Demographic and clinical characteristics

The demographic and clinical characteristics of 70 patients (mean age, 52.2 ± 13.8 years) are summarized in Table 1. There was no difference of mean age, CSF drainage volume, CSF opening/closing pressures, procedure time of LP, physician's experience of LP, number of LP attempts, CSF profiles, analgesics treatment, and disease diversity after LP between Group 1 and Group 2.

**Table 1:** Demographic and Clinical Characteristics of the subjects

	4 hours supine recumbence (n = 35)	1 hour supine recumbence (n = 35)	P Value
	**n(%) or Mean ± SD**	**n(%) or Mean ± SD**	
Sex, Female	14 (40%)	13 (37.2%)	0.806
Age (year)	51.4 ± 13.9	52.9 ± 13.8	0.644
Drainage amount (mL)	24.9 ± 11.0	22.8 ± 8.6	0.368
Opening pressure (cm H_2_0)	12.5 ± 4.3	13.5 ± 6.6	0.448
Closing pressure (cm H_2_0)	10.4 ± 5.7	10.4 ± 5.1	0.986
Experience of LP			
Yes	14 (40%)	11 (31.4%)	
No	21 (60%)	24 (68.6%)	0.509
Experience of PLPH			
Yes	4 (23.5%)	2 (20%)	
No	13 (76.5%)	8 (80%)	0.831
Physician			
Intern	1 (3%)	2 (6%)	
First-year Resident	33 (97%)	28 (85%)	0.138
Second-year Resident	0	3 (9%)	
LP attempts			
1	27 (77.1%)	25 (71.5%)	
2	4 (11.4%)	5 (14.2%)	
3	3 (8.6%)	4 (11.4%)	0.805
4	1 (2.9%)	0	
7	0	1 (2.9%)	
Leukocytosis in CSF (WBC > 5 mm^3^)	7 (20%)	7 (20%)	1.000
Treatment using analgesics after PLPH			
No use	4 (40%)	7 (58.3%)	
Non-opioids added	2 (20%)	1 (8.3%)	0.613
Non-opioids maintained	4 (40%)	4 (33.3%)	

LP = lumbar Puncture; PLPH = Post Lumbar Puncture Headache

### PLPH frequency in the two groups

Of 70 patients who underwent LP, 22 (31.4%) showed PLPH. The frequency of PLPH was not different between the two groups: 10 (28.6%) in Group 1 and 12 (34.3%) in Group 2 (P = 0.607, 95% CI: 0.474-3.590) (Table 2). The median onset time of PLPH was 16 hours (range 1-120 hours) after LP. PLPH occurred within 1 day in 17 patients (77.3%), within 2 days in 2 (9%), within 3 days in 2 (9%), and 120 hours later in 1 patient (4.5%). The median onset time of PLPH development did not significantly differ between groups (P = 0.582) (Table 2). The median severity of PLPH by NRS was 3 (range 1-10) in all patients and was not different between Group 1 (3, range 1-10) and Group 2 (2, range 1-9) (P = 0.203). Of 22 patients who had PLPH, 12 (54.5%) experienced PLPH within 1 day, 3 (13.6%) within 2 days, 3 (13.6%) within 3 days, and 2 (9.1%) within 5 days. The median duration of PLPH in all patients was 12 hours (range, 8-88 hours). The median duration of PLPH was not significantly different between groups (P = 0.521) (Table 2).

**Table 2:** Comparison of PLPH Frequencies in the 4 Hours and 1 Hour Supine Recumbence Groups

	4 hours supine recumbence (n = 35)	1 hour supine recumbence (n = 35)	P Value
	**n(%) or Median(range)**	**n(%) or Median(range)**	
PLPH			
Yes	10 (28.6%)	12 (34.3%)	0.607
No	25 (71.4%)	23 (65.7%)	
Onset time of PLPH (hour)	4.5 (range, 4-120)	20 (range, 1-56)	0.582
Severity of PLPH (NRS)	3 (range, 1-10)	2 (range, 1-9)	0.203
Duration of PLPH (hour)	28 (range, 8-88)	12 (range, 8-72)	0.521

PLPH = Post Lumbar Puncture Headache; NRS = Numeric Rating Scale

### Risk factors for the development of PLPH

The clinical characteristics of patients with and without PLPH in overall samples are summarized in Table 3. The frequency of PLPH was significantly higher in patients with previous history of PLPH (66.7%) than those without (16.7%) (P = 0.038). Patients with history of chronic headache prior to LP showed significant higher frequency of PLPH (85.7%) than those without (25.4%) (P = 0.003). In a logistic regression analysis, the previous history of PLPH was a significant risk factor for the development of PLPH (OR = 11.250, 95% CI: 1.10 - 114.369, P = 0.041).

**Table 3:** Clinical Characteristics According to the Presence of PLPH

	PLPH Yes (n = 22)	PLPH No (n = 48)	P Value
	**n (%) or Mean ± SD**	**n (%) or Mean ± SD**	
Sex, Female	7 (31.8%)	20 (41.7%)	0.598
Age (year)	50.7 ± 14.2	52.8 ± 13.7	0.545
Drainage amount (mL)	24.5 ± 12.9	23.6 ± 8.3	0.747
Opening pressure (cm H_2_0)	12.9 ± 5.1	13.0 ± 5.8	0.968
Closing pressure (cm H_2_0)	10.9 ± 4.3	10.2 ± 5.7	0.764
Experience of PLPH			
Yes	**4 (57.1%)**	**2 (11.8%)**	**0.038**
No	**3 (42.9%)**	**15 (88.2%)**	
Physician			
Intern	1 (5%)	2 (4.3%)	1.000
First-year Resident	18 (85%)	43 (91.4%)	
Second-year Resident	1 (5%)	2 (4.3%)	
LP attempts			
1	17 (77.3%)	34 (72.4%)	
2	3 (13.6%)	6 (12.9%)	1.000
3	2 (9.1%)	5 (10.5%)	
4	0	1 (2.1%)	
7	0	1 (2.1%)	
Leukocytosis in CSF (WBC > 5 mm^3^)	4 (18.2%)	10 (20.8%)	0.797
Chronic headache before LP			
Yes	**6 (27.3%)**	**1 (2.1%)**	
No	**16 (72.7%)**	**47 (97.9%)**	**0.003**

LP = Lumbar Puncture; PLPH = Post Lumbar Puncture Headache

Significant parameters are shown in bold.

Other factors such as age, sex, CSF pressure, CSF drainage volume, number of LP attempts, leukocytosis in CSF, or physician experience level was not associated with the development of PLPH (Table 3).

## Discussion

In the present study, there was no difference in the occurrence rate of PLPH between patients who had 1 hour of supine recumbence and those who had 4 hours of supine recumbence. These findings suggest that the duration of supine recumbence after LP may not play a key role in the development of PLPH. Therefore, the prolonged supine recumbence after LP may not be necessary to prevent PLPH.

The frequency of PLPH (31.4%) in the present study was similar to that of previous report with normal individuals or patients who underwent diagnostic LP[1,3]. In addition, the frequency of PLPH in this study was similar to that (36%) of previous studies using 22 gauge cutting needle[10,11]. The PLPH has been reported to occur more often in women than men[12,13]. This female predominance was also reported in patients with spontaneous CSF hypovolemia that is more complicated syndrome caused by spontaneous spinal dural tear without any trauma or LP[14,15]. However, there was no sex difference in the occurrence of PLPH in the present study. This sex-specific occurrence of PLPH needs to be further investigated.

Interestingly, patients with the previous history of PLPH showed significantly higher occurrence of PLPH than those without, which is in accordance with previous study[16,17]. In addition, patients with chronic headache before LP showed higher occurrence of PLPH than those without, which is also consistent with previous studies[3,5]. Increased frequency of PLPH in patients with previous history of PLPH or other types of chronic headache may reflect the certain underlying predisposition of this group of patients to developing PLPH.

The present study showed no correlation between PLPH frequency and age at study. This result is in agreement with a previous report[12]. The CSF volume, CSF flow dynamics, integrity of spinal dura matter may be affecting factors for the occurrence of PLPH according to age, but our study suggests that those factors are not essential.

There was no correlation between the total CSF volume loss (mean, 23.9 ± 9.8 mL; range 7-60 mL) and PLPH. Experimental study reported that orthostatic headache occurred when approximately 10% of the estimated total CSF volume was lost[18]. However, individual characteristics may also play a key role in the development of PLPH. These findings suggest that persistent CSF leakage after LP through an artificial hole in the spinal dura matter is more important factor than the absolute CSF volume loss during the procedure of LP.

The pathogenic mechanisms of PLPH may include downward pull on pain-sensitive structures caused by CSF volume loss, compensatory vasodilation of intracranial vessels in an attempt to maintain intracranial volume constant (Monro-Kellie doctrine) and hypersensitivity to substance P. Although the CSF volume loss caused by a large hole due to the manipulation of needle leads to decrease in intracranial pressure, the relationship between CSF pressure and the occurrence of PLPH is uncertain. In our study, the opening and closing CSF pressures were not associated with PLPH.

Our study had several limitations. First, our study was not carried out with randomization. Second, it was conducted in a single tertiary referral hospital. Third, several physicians performed LP. However, all physicians followed the standardized institutional guideline for LP. Fourth, sample size was small. However, priori power calculation showed that the sample size of this study was enough to have appropriate statistical power to interpret results. These limitations must be considered when the study findings are interpreted.

## Conclusions

Our study suggests that short duration (one hour) of supine recumbence may be as efficient as long duration (four hours) of supine recumbence to prevent PLPH.

## Abbreviations

LP: lumbar puncture; PLPH: post lumbar puncture headache; NRS: numeric rating scale.

## Competing interests

The authors declare that they have no competing interests.

## Authors' contributions

SRK contributed to the study design, data management, statistical analysis, data interpretation, and writing of the manuscript. SJC contributed to the study design, overall management, data interpretation, and manuscript editing.

HSC, MJY, JHH and KJC contributed to the data collection and data management. All authors contribute to the preparation of the manuscript and approved the final version submitted for publication.

## Pre-publication history

The pre-publication history for this paper can be accessed here:

http://www.biomedcentral.com/1471-2377/12/1/prepub
